# *Ectocarpus*: an evo-devo model for the brown algae

**DOI:** 10.1186/s13227-020-00164-9

**Published:** 2020-08-31

**Authors:** Susana M. Coelho, Akira F. Peters, Dieter Müller, J. Mark Cock

**Affiliations:** 1 CNRS, Sorbonne Université, UPMC University Paris 06, Algal Genetics Group, UMR 8227, Integrative Biology of Marine Models, Station Biologique de Roscoff, CS 90074, 29688 Roscoff, France; 2 Bezhin Rosko, Santec, France; 3 Fachbereich Biologie der Universitat Konstanz, 78457 Konstanz, Germany

**Keywords:** *Ectocarpus*, Life-cycle, Sex determination, Gametophyte, Sporophyte, Brown algae, Marine, Complex multicellularity, Phaeoviruses

## Abstract

*Ectocarpus* is a genus of filamentous, marine brown algae. Brown algae belong to the stramenopiles, a large supergroup of organisms that are only distantly related to animals, land plants and fungi. Brown algae are also one of only a small number of eukaryotic lineages that have evolved complex multicellularity. For many years, little information was available concerning the molecular mechanisms underlying multicellular development in the brown algae, but this situation has changed with the emergence of *Ectocarpus* as a model brown alga. Here we summarise some of the main questions that are being addressed and areas of study using *Ectocarpus* as a model organism and discuss how the genomic information, genetic tools and molecular approaches available for this organism are being employed to explore developmental questions in an evolutionary context.

## Natural habitat and life cycle

**Fig. 1:**
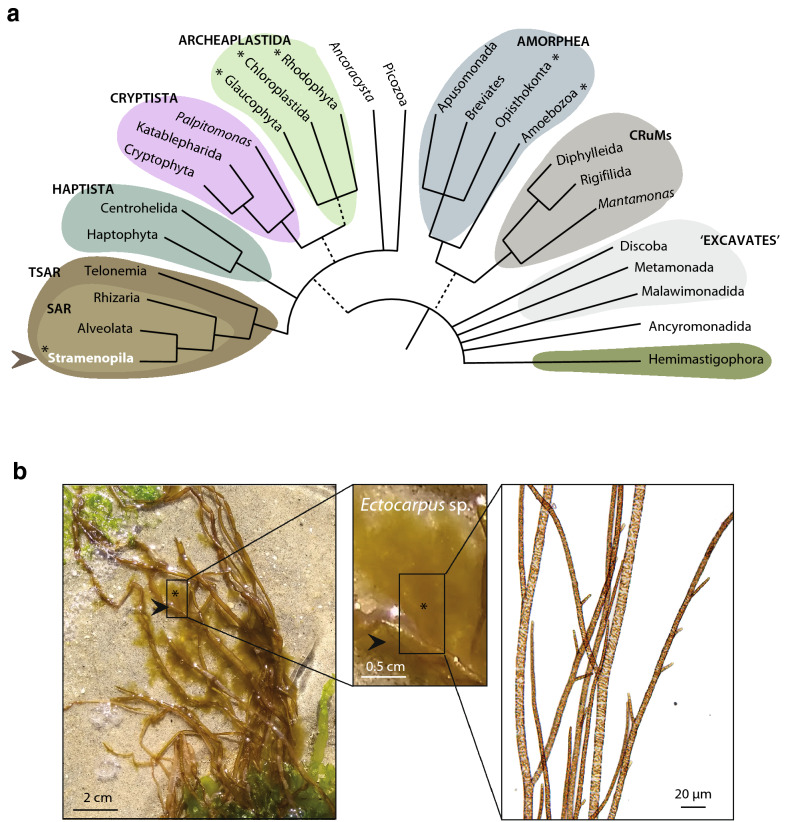
**a** Schematic view of the Eukaryotic tree, redrawn from [73]. The coloured groupings correspond to the currently recognised ‘supergroups’. Unresolved branching orders among lineages are shown as multifurcations. Broken lines reflect lesser uncertainties about the monophyly of certain groups. Asterisks represent lineages where complex multicellularity emerged. *Ectocarpus* is a brown alga, belonging to the Stramenopila (indicated with an arrowhead). **b** *Ectocarpus* sp. gametophyte (asterisk) in the field growing on the brown alga *Scytosiphon lomentaria* (arrowhead) (https://www.algaebase.org/search/species/detail/?tc=accept&species_id=76)

*Ectocarpus* is a genus of small, filamentous, multicellular, marine brown algae within the order Ectocarpales. Brown algae belong to the stramenopiles (or Heterokonta) (Fig. 1a), a large eukaryotic supergroup that is only distantly related to animals, plants and fungi. The stramenopiles include the macroscopic multicellular brown algae but also microbial algae (e.g., diatoms), diverse free-living heterotrophic or mixotrophic protists and important pathogens of animals and plants (e.g., *Blastocystis* or oomycetes). Brown algae or brown seaweeds are unique among stramenopiles (or heterokonts) in developing into multicellular forms with differentiated tissues, but they reproduce by means of flagellated spores and gametes that closely resemble cells of other heterokonts.

*Ectocarpus* is a cosmopolitan genus, occurring world-wide in temperate and subtropical regions, and has been collected on all continents except Antarctica [1]. It is present mainly on rocky shores where it grows on abiotic (rocks, pebbles, dead shells) and biotic (other algae, seagrass) substrata (Fig. 1b), and as a fouling organism it also colonises artificial substrata. It is found from the sublittoral to high intertidal pools, but it does not tolerate desiccation [2]. *Ectocarpus* sp. from Peru and northern Chile (SE Pacific) is the best studied species in the genus, and has become an established model for developmental biology and evolutionary questions (see below). While most species are exclusively marine, some species such as *Ectocarpus subulatus* may also occur in permanently brackish habitats such as the inner Baltic Sea and has even been encountered in mineral-rich freshwater [3].

Numerous taxa of *Ectocarpus* have been described since the creation of the genus [4]. However the taxonomy is complicated on one hand by oversplitting based on unreliable characters and on the other hand by the presence of cryptic species and hybrids. Sequence-based phylogenies have helped to disentangle the genus (e.g. [2, 5–7]) and molecular-assisted identification using nuclear (ITS) and cytoplasmic (COI, rbcLS spacer) barcode markers permit reliable identification. However, nomenclature is still incomplete because it is difficult to link described taxa to the lineages obtained in molecular phylogenies. Use of a combination of nuclear and mitochondrial markers has revealed the presence of natural hybrids [7, 8]. The existence of several species at different genetic distances and exhibiting differences in sexual compatibility, and the possibility of laboratory crosses [9] represent interesting options for genetic research.

**Fig. 2:**
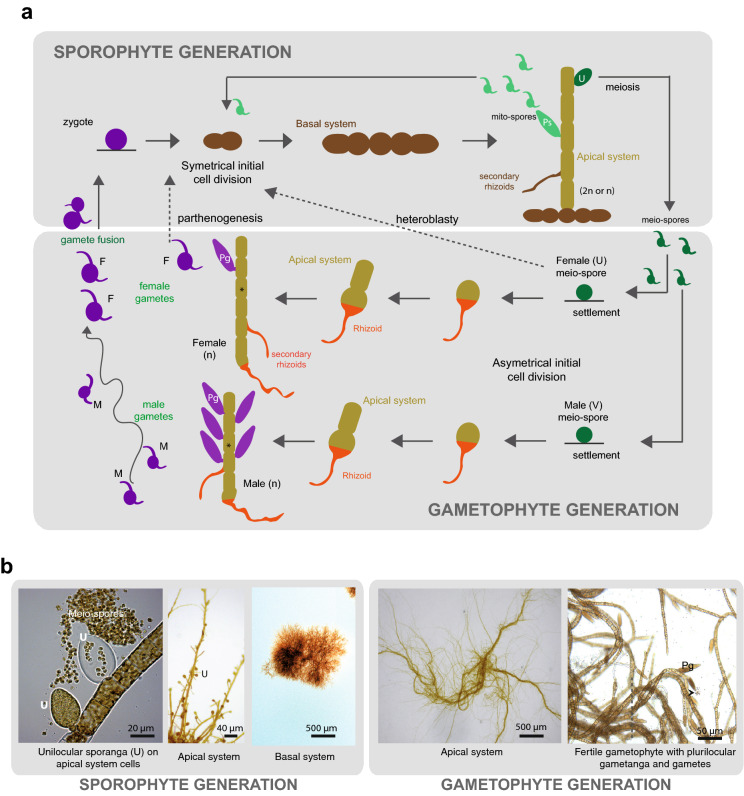
**a** Schematic view of the life cycle of *Ectocarpus* sp. Diploid sporophytes (ploidy 2n) produce plurilocular sporangia (Ps), where mito-spores are produced by mitosis. Mito-spores develop as clonal sporophytes. Sporophytes also produce unilocular sporangia (U), where meio-spores are produced via meiosis. Half of the meio-spores inherit a V sex chromosome and develop into male gametophytes, whereas the other half inherit a U sex chromosome and develop as female gametophytes. Male and female gametophytes (ploidy *n*) produce gametes at maturity in plurilocular gametangia (Pg). Male and female gametes are released into the surrounding seawater, where they fuse to produce zygotes that initiate the sporophyte generation. Gametophytes and sporophytes are subtly different in terms of cell types, cell size and angle of branching [16]. The *Ectocarpus* life cycle also includes several alternative pathways (dashed arrows). For example, if a gamete does not meet a gamete of the opposite sex, parthenogenesis may occur [17, 36, 74] and gametes develop into partheno-sporophytes, which are initially haploid (ploidy *n*) but may endoreduplicate to become diploid (ploidy 2*n*), allowing meiosis to occur [17]. In some *Ectocarpus* species, heteroblasty is common, and (haploid) meio-spores develop directly into haploid partheno-sporophytes. No difference has been observed between partheno-sporophytes arising from unfused gametes and partheno-sporophytes arising from heteroblasty (meio-spores). For simplicity, parthenogenesis and heteroblasty are illustrated for female gametes and meio-spores respectively, but in some strains male gametes and male meio-spores, respectively, may also go through these pathways. Details about the life cycle of *Ectocarpus* can be found in the literature [11, 15, 22, 74, 75]. Asterisk indicates cylindrical cells of the apical system. **b** Light micrographs of several stages of development of *Ectocarpus* sp. U: unilocular sporangium; Pg: plurilocular gametangium; Arrowhead: gametes

The *Ectocarpus* life cycle was first described in 1964 and 1967 [10, 11] using strains of *Ectocarpus siliculosus* from Naples, and later confirmed for other species (e.g. [12]). The sexual life cycle consists of an alternation between haploid male and female gametophyte and diploid sporophyte generations (a haploid-diploid life cycle with dioicy, Fig. 2). More details of the life cycle are given below.

## Laboratory culture and field collections

*Ectocarpus* is easily isolated in the laboratory from vegetative or fertile field material. Being a common alga, it is also regularly encountered as ‘contamination’ in isolates of other macroalgae, and is present as microstages on natural substratum, from which it may be isolated using the germling emergence technique. This technique consists of collecting substrata, incubating them in the laboratory and obtaining the alga as germlings that develop in culture [5]. In the field, the two generations may inhabit different niches and are often present at different seasons of the year. Different populations may employ sexual and asexual reproduction to different degrees [13]. Culture in Petri dishes or in large flasks (when a large amount of material is needed, e.g. for protein or nucleic acid extractions) using standard seawater medium complemented with nutrients is straightforward [13]. Gametophytes and sporophytes are grown under similar culture conditions and the full life cycle can be completed in the laboratory in about 2 months [13]. Standard growth conditions are 13 °C with a 12 h/12 h day/night cycle and 20 μmol photons m^−2^ s^−1^ irradiance [13]. Strains may grow axenically and can be stored under low light and low temperature conditions for at least one year before the culture needs to be refreshed [13]. In addition, an alternative stock maintenance method based on cryopreservation is also available [14].

## Major interests and research questions

The genus *Ectocarpus* has been studied for many years [1] and the species *Ectocarpus* sp. [7] from the SE Pacific [8] is currently the model of choice for the application of genomic and genetic approaches to diverse questions concerning the biology of the brown alga. Some examples are given below.

### Life cycle

Like many brown algae, *Ectocarpus* has a haploid–diploid life cycle that involves alternation between two multicellular generations, the sporophyte and the gametophyte (Fig. 2; [10, 15]). Both generations consist of uniseriate, branched filaments, but there are some morphological differences between the two generations [15, 16]. Gametophyte germlings are made up of a rhizoid and an upright filament, the latter consisting of cylindrical cells (Fig. 2). The upright filament grows and profusely branches to produce the mature thallus, which carries plurilocular gametangia, the reproductive structures in which gametes are produced. The developmental programme of the sporophyte is slightly more complex in that it produces a basal system consisting of round and elongated cells before producing the upright filaments of the apical system (Fig. 2). The upright filaments of the sporophyte resemble those of the gametophyte but are less branched, and the angle of branching (about 90°) is larger than in gametophytes ([16]; Fig. 2b). Sporophyte upright filaments produce two types of reproductive structure, plurilocular sporangia containing mito-spores (which germinate to produce clones of the parent sporophyte) and unilocular sporangia in which a single meiotic division followed by several mitotic divisions produces the meio-spores (the initial cells of the gametophyte generation) (Fig. 2).

*Ectocarpus* sporophytes can be derived from zygotes (i.e. formed by the fusion of two gametes to produce a diploid individual) or can develop parthenogenetically from a gamete that has failed to find a partner of the opposite sex (in which case they are called partheno-sporophytes). Being derived from a single gamete, most partheno-sporophytes are haploid (Fig. 2, but see [17]). Note that there are no conspicuous morphological differences between a diploid (zygote-derived) sporophyte and a partheno-sporophyte. Parthenogenesis has been exploited to isolate life cycle mutants using UV mutagenised gametes, where screens were designed to identify individuals that deployed the gametophyte instead the sporophyte developmental programme [18]. This approach led to the identification of two loci involved in the alternation of generations, *OUROBOROS *and *SAMSARA*. The *ORO* and *SAM* genes encode TALE-homeodomain transcription factors (TALE-HD TFs). TALE-HD TFs are involved in life cycle regulation in green algae [19] and also control sporophyte to gametophyte transitions in land plants [20]. It appears therefore that there has been a convergent recruitment of TALE homeodomain life cycle regulators to direct sporophyte development in land plants and brown algae [21].

The *Ectocarpus* sporophyte generation secretes a diffusible factor into the medium that can induce gametophyte initial cells (meio-spores) to switch to the sporophyte developmental pathway [22]. Switching to the sporophyte generation is only observed if meio-spores are treated with sporophyte-conditioned medium (containing the diffusible factor) before they start to synthesise a cell wall. The diffusible factor appears to act upstream of *ORO* and *SAM* because both *oro* and *sam* mutants are insensitive to treatment with the factor [18, 23].

### Organelle inheritance

Organelle inheritance is usually strictly regulated during the transition from the haploid to the diploid phase of the life cycle to avoid cytoplasmic conflicts and to limit the spread of selfish genetic elements (reviewed in [24]). In *Ectocarpus*, plastids are biparentally inherited but the paternal and maternal plastids segregate to different sets of daughter cells during early development, resulting in chimeric thalli in which cells inherit either the paternal or the maternal organelle [25, 26]. Mitochondrial inheritance is usually maternal [26] but a recent study has identified unusual patterns of mitochondrial inheritance in some strains of *Ectocarpus* [27].

### Developmental patterns and the evolution of multicellularity

The transition to complex multicellularity is a major evolutionary event, and has arisen rarely during eukaryotic evolution [28]. The brown algae are one of only five eukaryotic lineages that have evolved this characteristic (the four others being animals, green plants/algae, fungi and red algae). Each of these lineages has independently evolved the developmental control mechanisms needed for the construction of a complex multicellular organism. In animals and land plants, the molecular basis of many developmental processes is well understood but very little is known about these processes in the other three lineages. In recent years, *Ectocarpus* has been used to study the molecular basis of development in the brown algae.

*Ectocarpus* exhibits a relatively simple pattern of development, with a small number of different cell types [29]. Ultraviolet irradiation has been used to generate developmental mutants, and the affected genes have been identified by classical genetic analysis [30]. Note that the possibility of obtaining both the gametophyte and sporophyte generations as haploid individuals greatly facilitates genetic analysis because phenotypes are readily detectable. In other words, recessive phenotypes are visible in the haploid phase and there is no requirement for back-crosses, in contrast with diploid model organisms where genetic crosses are necessary*.*

Several mutants have been employed to understand developmental pattern formation in *Ectocarpus*. In the *immediate upright* (*imm*) mutant, the initial cell undergoes an asymmetrical (instead of symmetrical) division to immediately establish the apical-basal axis [15, 30]. The affected gene encodes a protein of unknown function that contains a repeated motif also found in the EsV-1-7 gene of the *Ectocarpus* virus EsV-1. The EsV-1-7 gene family has expanded in the brown algae compared with unicellular outgroup lineages, and it has been proposed that this expansion may be linked with the emergence of multicellular complexity. EsV-1-7 domain genes have a patchy distribution across eukaryotic supergroups and occur in several viral genomes, suggesting possible horizontal transfer during eukaryote evolution [30].

Mutations at the *IMM* locus only affect the sporophyte generation of *Ectocarpus*, suggesting that this gene controls developmental patterns specific to the sporophyte. Other loci, however, operate during both generations. Mutations in the *Ectocarpus DISTAG* (*DIS*) gene lead to loss of basal structures (round cells and rhizoids in the sporophyte generation; rhizoids in the gametophyte generation; Fig. 2) during both the gametophyte and the sporophyte generations. Consequently, *dis* individuals are free floating and composed only of apical system cells. Several abnormalities were observed in the germinating initial cell in *dis* mutants, including increased cell size, disorganisation of the Golgi apparatus, disruption of the microtubule network, and aberrant positioning of the nucleus. *DIS* encodes a TBCCd1 protein. TBCCd1 has a role in cell organisation in animals, *Chlamydomonas reinhardtii*, and trypanosomes [31–33]. Studies using *Ectocarpus* have thus emphasised the conservation of TBCCd1 function in the regulation of cellular structures across extremely distant eukaryotic groups.

*Ectocarpus* has also been used to investigate auxin function in morphogenesis across eukaryotes [34] and as a comparative model organism to understand general principles on branching morphogenesis (reviewed in [35]).

### Sexual reproduction, evolution of sexes and sex chromosomes

As described above, *Ectocarpus* has separate male and female gametophytes. Male and female gametes produced by each sex are both flagellated but differ with respect to their size, physiology and behaviour: female gametes are slightly bigger, settle sooner and produce a pheromone whilst male gametes swim for longer and are attracted to the pheromone produced by the female. The pheromone that attracts male gametes is an unsaturated hydrocarbon [36, 37] and there is evidence that cell-to-cell recognition between gametes is mediated by N-acetyl glucosamine residues exposed on the plasma membrane of female gametes that are recognised specifically by a receptor on the male gamete [38].

Sex in *Ectocarpus* sp. is determined during the haploid phase of the life cycle by a pair of U/V sex chromosomes [11, 39, 40]. *Ectocarpus* sp. has been used as a model to study the genomic features of U/V sex chromosomes. These chromosomes arose early during the evolution of the brown algae, at least 150 MY ago [39, 41, 42]. A conserved gene encoding a High Mobility Group (HMG) domain protein was found within the V (male)-specific region of the sex chromosome in *Ectocarpus*, and comparative analysis across several other brown algal species showed that orthologs of this gene are consistently male-linked. This result, together with the fact that the HMG gene is strongly upregulated at fertility, makes it an interesting candidate for a master male sex determination gene.

*Ectocarpus* has also become a reference for studies of the mechanisms underlying sexual differentiation during haploid dioicy (separate sexes in the haploid phase of the life cycle) [43–45]. Comparative RNA-seq analysis using male and female isogenic lines identified several hundred genes that were differentially regulated in male compared to female gametophytes [44].

### Response to stress

Microarray analysis has indicated that the expression patterns of a large proportion (70%) of *Ectocarpus* genes are modified in response to various abiotic stresses [46]. A genetic approach has identified 39 quantitative trait loci that influence the growth response in response to temperature and saline stress [47]. The resources established by these analyses provide a starting point both for understanding adaptation of brown algal populations to different environments, for example in response to climate change, and for the application of breeding approaches in brown algae to improve growth yields under different environmental conditions.

### Symbiotic relationships

*Ectocarpus* has been shown to interact with a range of other organisms in relationships that range from commensal (or possibly mutualistic) to pathogenic. Reported pathogens include viruses, oomycetes, chytrids and the plasmodiophorean *Maullinia ectocarpii* [48]. *Ectocarpus siliculosus* Virus 1 (EsV-1) is a large DNA virus that is capable of integrating into its host genome [49]. The integrated virus has been shown to segregate to half of the progeny following meiosis [49]. The virus can therefore propagate both through the production of infective virus particles and vertically by genetic inheritance. The complete sequence of EsV-1 showed that it has a large genome of 335 kbp containing 231 genes [50]. Virus infections appear to be very prevalent in filamentous brown algae such as *Ectocarpus* [51] and strains may contain viral DNA without exhibiting viral symptoms [52].

*Ectocarpus* has also been shown to associate with a broad range of bacterial species [53] and there is evidence that this microbiome can influence the development of the alga [54].

## Experimental approaches

### Mutant analysis

Protocols have been developed for both ultraviolet and chemical (EMS and ENU) mutagenesis along with screening methodologies for developmental mutants [23, 55]. Thousands of individuals placed in petri dishes or in 96-well plates can be individually followed and easily screened under a microscope or stereomicroscope. Classical genetic analysis of mutants is possible, with protocols having been developed both for carrying out genetic crosses and for the isolation of meiotic progeny [9, 10, 13, 56]. Using these approaches large segregating populations can be generated for mapping experiments [30] and mutant alleles have also recently been identified by a cloning-by-sequencing approach [16].

### Immunocytochemistry

*Ectocarpus* has relatively small cells (10–30 µm) and the uniseriate filaments can be grown in two dimensions directly attached to the surface of microscope slides. These features make *Ectocarpus* particularly suitable for high resolution imaging approaches, specifically after fixation and clarification. Optimised protocols for the detection of tubulin are available [57] but may be used with any suitable antibody. Immunostaining protocols have been employed successfully to describe the cellular modifications associated with mutations in *Ectocarpus* genes involved in pattern formation (e.g. [16]). Immunostaining using specific monoclonal antibodies directed against sulphated cell wall polysaccharides has also been employed to investigate cell wall dynamics during early development of *Ectocarpus* [58] and to describe cellular responses to osmotic stress [59].

### Protoplasts

Protoplasts can be obtained by enzymatic digestion of *Ectocarpus* filaments of both the gametophyte and sporophyte generations. The resultant protoplasts are totipotent and regenerate to produce individual thalli under appropriate culture conditions [60]. The naked, wall-less cells produced can be used for several applications, including studies of cell wall regeneration, investigation of the role of the cell wall in determining cell fate [21, 22], and as a source of naked cells for the development of methods for introducing diverse molecules into the cell, including transfection.

### Live imaging

The fact that *Ectocarpus* cells grow attached to a substratum makes cells easily accessible to live imaging experimentation, at least during the early stages of development before three-dimensional growth is initiated. Filaments of *Ectocarpus* have been used as model systems to study morphogenesis and mechanical proprieties of cells along a filament [61, 62]. Quantitative measurements at the cellular level and biophysical simulation approaches have allowed tip growth mechanisms in *Ectocarpus* to be investigated using time lapse imaging, and live imaging techniques such as fluorescence recovery after photobleaching (FRAP) highlighted active vesicle trafficking in the shanks of the apical cell [61]. *Ectocarpus* is also amenable to pulse-chase experiments using cell wall dyes such as Calcofluor White, which can be used to follow the position and direction of cell wall expansion during growth.

*Ectocarpus* gametes have been used as models for flagella movement and swimming behaviour in response to light and sex pheromones, by employing live imaging and high-speed video cameras (e.g. [63, 64]).

One important challenge in imaging *Ectocarpus* is the auto-fluorescence of some tissues, especially older filaments. This feature will need to be taken into consideration in the future when choosing fluorescent proteins for transfection.

### Single-cell techniques

Laser capture microdissection (LCM) facilitates the isolation of individual cells from tissue sections, and when combined with RNA amplification techniques, it is an extremely powerful tool for examining genome-wide expression profiles in specific cell-types. LCM has been used to study the cell-specific transcriptome of *Ectocarpus* sporophytes [65]. Future progress in cell-specific approaches would be valuable and, in combination with mutant characterisation by genetic and genomic techniques [16], will increase our understanding of the processes that underpin body-plan specification and allow a mechanistic understanding of *Ectocarpus* development.

### Epigenetics

Recent progress in the development of chromatin extraction protocols for *Ectocarpus* have allowed the investigation of histone post translational modifications [66]. This methodology is currently being used to investigate the role of epigenetic processes during life cycle progression and sexual differentiation.

### Reverse genetics approaches

The major bottleneck for *Ectocarpus* as a model system has been the lack of tools to investigate gene function, and it has proved to be very difficult to develop a reliable genetic transformation protocol for this model organism. Protocols have been developed for the delivery of reagents by biolistics and microinjection but, to date, no transgene activity has been detected in *Ectocarpus*. Optimising transgene structure may be one of the bottlenecks, given the unusual features of endogenous *Ectocarpus* genes (abundant introns, short intergenic regions; [67]). Gene silencing (RNA interference‐mediated gene knockdown) has been developed for the brown alga *Fucus* [68], and has been recently used to analyse gene function in *Ectocarpus* [16, 30]. Current efforts are aimed at adapting the CRISPR-Cas9 system for use in *Ectocarpus*.

## Research community and resources

### *Ectocarpus* culture collections

There are considerable genetic resources available for *Ectocarpus*, including a collection of more than 2000 strains held at the Station Biologique de Roscoff, some of which are available through the Roscoff Culture Collection (RCC), France. These strains represent the worldwide diversity within the genus, but also include several collections of populations from single sites, providing access to information about local population structures and diversity. *Ectocarpus* strains are also kept at the Culture Collection of Algae and Protozoa (CCAP) at the Scottish Association for Marine Science and at the Kobe University Macroalgae Culture Collection (KU-MACC) in Japan.

### *Ectocarpus* and brown algae International Meetings

The *Ectocarpus* research community currently comprises about ten laboratories worldwide, but several dozen laboratories use *Ectocarpus* together with other model systems for comparative analysis. *Ectocarpus* researchers have met approximately every 2 years since 2010 for 3-day International *Ectocarpus* Meetings, which all researchers interested in initiating research on *Ectocarpus* are welcome to join. Moreover, members of the *Ectocarpus* research community also regularly attend European and International Phycological Congresses. The Phaeoexplorer project (https://www.france-genomique.org/projet/phaeoexplorer/) is assembling more than 40 laboratories from across the world interested in several aspects of *Ectocarpus* and brown algal biology.

### Genome, transcriptomes and genome browsers

The small size of the *Ectocarpus* genome (214 Mbp; [67]) compared to those of most other brown algal species represented an asset in the choice of this organism as a model for the brown algae. The complete high quality sequence of a reference genome is available and the transcribed regions have been characterised using Sanger ESTs, microarrays [46] and RNA-seq datasets from several stages of development during the life cycle [30, 46, 69, 70]. Genome data and genetic data have been combined to generate a sequence-anchored genetic map [71] that has been used for genetic analysis e.g. [30] and more recently, a very high quality assembly and a Hi-C physical map has been generated [47, 69, 72]. Currently, the *Ectocarpus* genome browser is hosted at the VIB, Belgium (https://bioinformatics.psb.ugent.be/orcae/overview/EctsiV2).
